# Distance-based Device-To-Device outage reduction for 5G wireless systems

**DOI:** 10.1371/journal.pone.0335050

**Published:** 2026-06-15

**Authors:** Abdullah Hadi Al-Quhali, Mardeni Roslee, Mohamad Yusoff Alias, Osama Abu Ajwa

**Affiliations:** 1 Center of Wireless Technology (CWT), Multimedia University, Cyberjaya, Selangor, Malaysia; 2 Faculty of Engineering (FOE), Multimedia University, Cyberjaya, Selangor, Malaysia; 3 Telekom Malaysia R&D, Multimedia University, Cyberjaya, Selangor, Malaysia; Jaramogi Oginga Odinga University of Science and Technology, KENYA

## Abstract

Device-to-Device (D2D) communication constitutes that closely spaced nodes communicate with each other without the use of a centralized base station that carries the communication data. This helps in increasing the data rate, decreasing latency, and expanding the bandwidth and capacity which promotes it to play an important part in next-generation wireless technology. Regardless, the efficient utilization of the available resources is a crucial challenge in D2D systems especially in limited areas; considering that the spectrum is shared across the cell. This paper investigates the spectrum sharing strategies for D2D communication in the context of a single cell by presenting a simulation study of D2D communication performance under different resource allocation scenarios. The first scenario divides the coverage area into inner and outer regions, and the spectrum as well; one for each region, the second scenario assigns resources to nearby users within a range of 10 meters and reuses the resources by other distant users if the separation is more than 50 meters. The variations in the simulation parameters helps to analyze the impact of user location, density, and resource allocation in outdoor urban environments. The simulation results are evaluated in terms of spectrum utilization, and outage probability. These results are analyzed to understand the impact of resource allocation on the performance of the system and thereby show that the second scenario improves the system performance in terms of the outage probability providing useful insights for designing and optimizing D2D communication systems in an outdoor urban environment especially in next-generation-networks.

## Introduction

Over the past few years, wireless communication has brought significant enhancements to the user experience by providing convenient, and flexible access to their information through multiple communication services. Thanks to the wireless technology, users are able to communicate with others from any location, as long as they are within range of a wireless network [1]. This eliminates the need for users to be physically connected to a network via cables, which can be cumbersome and inconvenient. Additionally, wireless communication allows users to connect multiple devices to a network, such as smart-phones, laptops, tablets, and smart home devices, allowing them to exchange and share information with others using a variety of devices. Furthermore, wireless communication enables users to collaborate and work together in real-time, regardless of their location, improving the efficiency and effectiveness even in remote and rural locations [2].

Despite the undeniable advances in wireless communication, traditional cellular systems are increasingly limited the weight of rapidly growing user demands, devices growth, and spectrum scarcity. Such systems face inherent limitations that fundamentally hinder scalability and performance in next-generation networks. Specifically, issues such as proximity transmission, susceptibility to interference, limited spectrum capacity, and increased vulnerability to security threats including eavesdropping, jamming, and power leakage pose critical challenges to reliable and efficient wireless communication [3].

As communication systems transition toward ultra-dense networks characteristic of 5G and beyond, these constraints become even more evident. The rise in base station deployments and user density increase the strain on centralized infrastructure, making it clear that conventional network models are no longer sufficient to meet the latency, coverage, and capacity requirements of modern applications. To address these growing demands, D2D communication emerges as a transformative paradigm. By enabling direct communication between user devices, bypassing the base station for appropriate services, D2D significantly enhances spectrum utilization, reduces latency, and improves energy efficiency. This paradigm not only reduces network congestion but also opens new doors for proximity-based services, emergency communication frameworks, and context-aware content delivery [4].

Thus, the limitations of conventional cellular architectures, when added to the evolving expectations of modern wireless systems, necessitate the exploration and optimization of D2D communication mechanisms. This study builds upon this urgency by investigating spectrum sharing strategies in D2D contexts, aiming to establish robust, efficient, and scalable solutions for future wireless ecosystems [5].

D2D also opens up new possibilities for proximity-based commercial services such as professional networking applications and load balancing services [6–8] public safety, local data transfer, data overflow, and network cryptography [9]. In addition, D2D offers advantages such as increased spectrum efficiency, wider cellular coverage, higher energy efficiency, and reduced back-haul demand [10,11].

**Fig 1:**
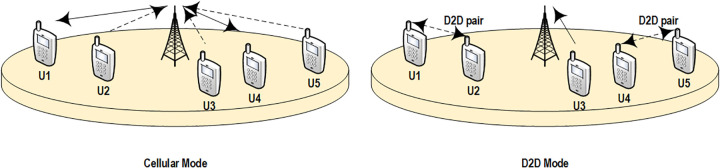
Cellular Vs. D2D.

In recent years, the trend toward proximity-based services and the need for public safety services have fuelled researchers to focus their attention on the use of D2D networks, or, more broadly, the concept of a hybrid network that combines infrastructure-based and ad-hoc networks [12,13]. These works assess the ability of mobile devices to relay radio signals in cellular networks as well as their effectiveness to improve coverage, throughput, and overall cellular network performance [9]. These researches are being pursued in parallel with the industry’s standardization effort in order to overcome the many fundamental difficulties related with enabling D2D in cellular networks. One of the most fundamental concerns is how to share spectrum resources between cellular and direct access connections as illustrated in Fig 1. This identifies that D2D can be classified into two types based on the type of spectrum sharing employed. The most common type of spectrum sharing is in-band spectrum sharing, which refers to D2D that uses the cellular spectrum, whereas out-band D2D uses bands other than the cellular spectrum (for example, the 2.4 GHz ISM band). D2D is further subdivided into two categories: overlay and underlay whereby in the overlay, both cellular and D2D transmitters can use orthogonal time/frequency resources, whereas underlay implies that D2D transmitters opportunistically access the time/frequency resources held by cellular users [14].

**Fig 2:**
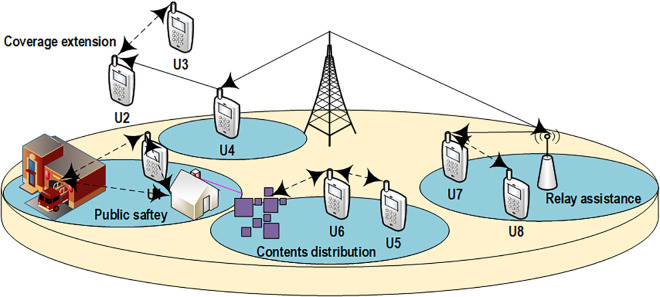
D2D Modes.

Generally, D2D spectrum sharing bypasses the cellular network, as opposed to cognitive radio networks, where spectrum sharing is done by the cognitive radio network itself. Regardless of this, the question of how D2D should access the spectrum remains a challenge due to the additional complications of D2D mode selection, which implies that a prospective D2D pair may switch between direct and conventional cellular connections [1]. Additionally, D2D networking differs from traditional ad-hoc networking because of its ability to use cellular network infrastructure that a traditional ad-hoc network cannot. D2D communication can also be used in different modes, such as direct mode, relay mode, and network-assisted mode [15]. In direct mode, the D2D pair communicates directly with each other using their own radio resources. In relay mode, one of the D2D devices acts as a relay to forward the data between the other D2D device and the cellular network. In network-assisted mode, the D2D devices communicate with the cellular network, which then relays the data between the D2D devices as shown in Fig 2.

Despite the enhancements that the D2D promises, it can also cause interference to the cellular users, especially when the D2D pairs are in close proximity to each other or to the cellular base station [16]. To mitigate this interference, various resource allocation algorithms have been proposed, such as distance-based resource allocation [17], joint power and channel allocation [16], and energy-efficient resource allocation [18] algorithms which aim to maximize the spectral efficiency or energy efficiency of the D2D communication while minimizing the interference caused to the cellular users. Additionally, power control in [19], rate adaptation in [20], and scheduling in [21] algorithms that aim to optimize the performance of D2D communication under different scenarios and conditions.

It can be concluded that the performance of D2D communication depends on various factors, such as the distance between the D2D devices, the transmit power of the user equipments, the channel conditions, and the interference caused by other devices. Therefore, it is important to carefully design the resource allocation algorithm to maximize the spectrum efficiency of the D2D communication while minimizing the interference caused to the cellular users [22].

This paper describes existing research relevant to spectrum sharing mechanisms in radio networks, and subsequently presents a simulation for a D2D environment using Matlab tool, proposing a spectrum sharing method that enables better spectrum efficiency, throughput, and signal to interference plus noise ratio (SINR), It then proposes a distance-based D2D spectrum utilization as a way to improve the use of radio spectrum in wireless networks by allowing devices that are distanced apart to communicate directly. This can be helpful in 4G and 5G wireless technology, where there is a high demand for fast data transfer and a limited amount of available spectrum. In that regard, the proposed method can be beneficial in safely offloading traffic from the cellular network, improving the spectrum efficiency, and enhancing the coverage of the wireless network. The following are the most significant contributions of this article.

- A comprehensive evaluation of the performance of D2D communication under different resource allocation scenarios which allows for a better understanding of the impact of user and resource allocation on the performance of the D2D communication system in an outdoor urban environment.
- The simulation results show that the second scenario, where resources are assigned to nearby users, improves the system performance in terms of the outage probability which suggests that the dynamic allocation of resources to nearby users can be an effective approach to improve the overall system performance in high-density scenarios.
- A realistic simulation scenario that can be used as a reference for future studies and can provide useful insights for designing and optimizing D2D communication systems in such environments.

The rest of this paper is organized as follows, Section describes the next-Generation-networks, the related works are presented in Section, the experiment modelling and setup are presented in Section. Section discusses the results of the experiments followed by the concluding remarks in Section.

## Device-to-Device background

New technologies, particularly in wireless communication and mobile computing, are fundamentally transforming how individuals share information. Regardless, mobile users’ connectivity is limited by base station (BS) coverage and does not allow for direct communication between mobile devices [23]. New information and communication systems should be able to exchange data on-demand while maintaining and expanding network capacity. D2D communications are viewed as a viable solution for allowing mobile devices to communicate directly with one another without the use of access points or BSs [4].

The goal of D2D in next-generation networks is to improve signal quality for mobile devices in a sparse environment by leveraging the physical range of communication devices. D2D communication and cellular networking services should be used in conjunction to support one another. In general, it is critical to consider resource sharing before implementing the use of D2D in cellular communication [23].

### Overlay versus underlay

In a D2D communication network, the overlay and underlay approaches refer to the way in which D2D communication is implemented in relation to the existing cellular network. In the overlay approach, the communication is implemented as an additional layer on top of the existing cellular network while it uses its own separate radio frequency spectrum and does not interfere with the operation of the cellular network. The advantage of the overlay approach is that it allows for a more flexible and efficient use of the available spectrum resources. However, it requires additional infrastructure and resources to support the D2D communication layer [12].

On the other hand, the underlay approach forces the implementation of communication as a secondary layer that operates within the same radio frequency spectrum as the cellular network. In this approach, D2D communication coexists with the cellular network and may potentially interfere with its operation. To mitigate this potential interference, careful resource allocation and interference management techniques are required. The advantage of the underlay approach is that it can be implemented with minimal additional infrastructure and resources, as it leverages the existing cellular network infrastructure. However, it may not be as efficient in terms of spectrum utilization compared to the overlay approach.

**Fig 3:**
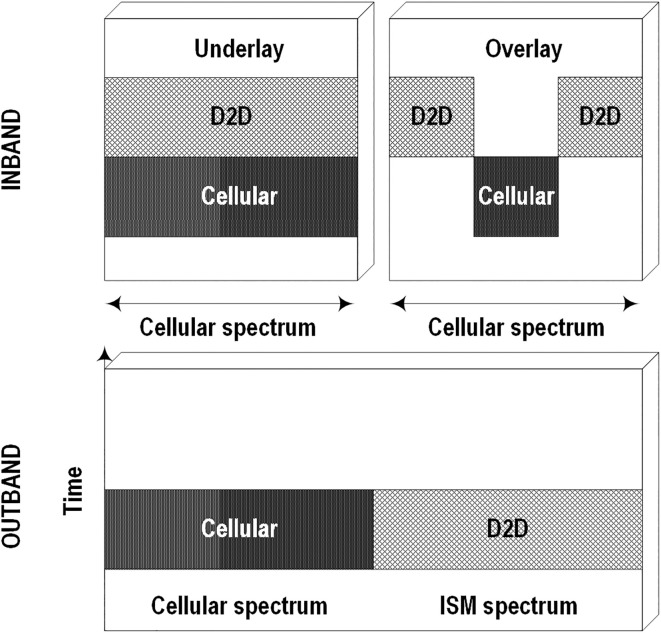
Underlay Vs. Overlay.

It is understood that both approaches as illustrated in Fig 3, have their own advantages and disadvantages, and the choice of which approach to use may depend on the specific requirements and constraints of the communication network.

Table 1 describes the differences between overlay and underlay structures in cellular network communication whereby in both cases, the base station or access point is responsible for coordinating the communication between the D2D users and the cellular network, and for managing the allocation of resources such as radio frequency spectrum or power.

**Table 1:** Overlay and Underlay Structures.

Entity	Overlay	Underlay
Base station	responsible for communicating with the D2D users and forwarding their data to the cellular network	responsible for communicating with both the cellular users and the D2D users, and forwarding their data to the cellular network
D2D user	communicate with other D2D users or directly with the base station/access point	uses D2D connection to communicate with the base station/access point
Cellular user	–	uses the cellular network to communicate with other devices or with the Internet
Radio frequency spectrum	separate spectrum band is dedicated for D2D communication, which is different from the spectrum band used by the cellular network	D2D communication operates within the same radio frequency spectrum as the cellular network

To summarize, the main difference between the overlay and underlay approaches is the way in which D2D communication is implemented in relation to the existing cellular network.

## Next-generation-networks and spectrum-related challenges

One potential application of D2D communication in next-generation networks is the use of D2D communication to offload traffic from the cellular network on the contrary of maximizing the base stations because of the short ranges of the higher frequencies. For example, if two devices are in close proximity to each other and both have a strong cellular connection, they could use D2D communication to exchange data rather than using the cellular network. This would reduce the load on the cellular network and potentially improve the overall performance of the network. Another potential application is the use of D2D communication to improve network coverage in areas where the cellular network coverage is weak or unavailable to establish communication between devices by creating a mesh network using the devices themselves as routers. This can help to extend the reach of the network and improve connectivity in areas where it would otherwise be difficult to do so.

More examples of D2D communication might be used in next-generation networks include:

- **Enhancing public safety communications:** it can be used to improve communication in special areas, such as fire-zones and crime scenes, or in emergency situations where traditional communication infrastructure may be unavailable.
- **Enabling peer-to-peer communication:** used to enable direct communication between devices without the need for a central station or infrastructure. This could be used for applications such as file sharing or video conferencing.
- **Improving energy efficiency**: D2D communication can be used to reduce the power consumption of devices by allowing them to communicate directly with each other rather than relying on a central network infrastructure. This can be especially beneficial for devices with limited battery life, such as internet of things (IoT) devices.

One of the main challenges in implementing D2D communication is the limited availability of spectrum resources that must carefully be designed and allocated in order to ensure efficient and effective communication. In most cases, D2D devices communicate directly with each other using a shared spectrum band which can lead to interference and reduced capacity if the spectrum band becomes overcrowded. Additionally, D2D communication often operates in unlicensed spectrum bands, which are not dedicated to any specific user or service making it more difficult to ensure reliable and consistent communication, as other users or devices may be using the same spectrum band at the same time [24,25].

To address these challenges, advanced techniques such as spectrum sharing and dynamic spectrum management are being developed to improve the efficiency and capacity of D2D communication. These techniques allow devices to adapt to changing spectrum conditions and make more efficient use of the available spectrum resources. However, implementing these techniques can be a complex problem and requires careful planning and coordination.

In [26], a review is conducted to discusses the challenges and opportunities of using D2D communication in 5G and future networks. The authors describe various challenges, such as interference, security, and energy efficiency, and discuss how they can be addressed through advanced techniques such as spectrum sharing, dynamic spectrum management, and network coding. The authors also discussed the opportunities of using D2D communication in various scenarios, such as emergency communication, public safety, and IoT applications, and provide an overview of the current state of the art in this field. More works on the highlighted challenges are summarized in the subsequent section.

## Related works

The authors of [16] proposes a joint power and channel allocation algorithm for D2D communication in cellular networks, which aims to maximize the spectral efficiency while taking into account the interference caused by D2D communication. The algorithm is based on a game-theoretic approach and is designed to maximize the sum rate of the D2D pairs while minimizing the interference caused to the cellular users.

In [27], the authors proposed an energy-efficient resource allocation algorithm for D2D communication in cellular networks, which aims to minimize the energy consumption while maximizing the system throughput.

The authors of [18] proposed a distance-based resource allocation algorithm for D2D communication in cellular networks, which takes into account the distance between devices and the interference they cause to each other. The algorithm is based on the concept of opportunistic D2D communication, which allows devices to communicate directly with each other when they are in close proximity and the interference caused to other devices is minimal.

The study in citewang2015outage, presentes a well-structured analytical framework that addresses a crucial aspect of D2D communications, it studies the impact of co-channel interference on both D2D and conventional cellular (CC) transmissions, particularly in multi-hop scenarios. By employing the shortest path routing (SPR) algorithm, the authors derive closed-form expressions for key performance metrics such as the number of hops and outage probability, offering both theoretical and numerical validation. The authors quantified the reliability of D2D links, which remain robust with an outage probability below 5%, while simultaneously highlighting the significant degradation caused to CC transmissions, where the outage exceeds 25%.

The survey in [28] provides an overview of D2D communication in cellular networks and discusses the challenges and opportunities presented by this technology. The authors review different D2D communication scenarios, such as in-band D2D, out-of-band D2D, and spectrum-sharing D2D, and highlight the key technical challenges and open research issues in each scenario.

In [29], the authors investigate the use of distance-based resource allocation for D2D communication in millimeter wave cellular networks, which have the potential to offer high data rates but are also susceptible to interference.

## Modeling

To assess the performance of D2D communication under different resource allocation scenarios, a simulation study is developed using Matlab with the modelling and evaluation of different communication scenarios. It considers three main scenarios: proposed D2D scenario, conventional cellular, and hybrid. In these scenarios, a certain number of users participate in the communication process. Spectrum allocation is performed, where a portion of the available spectrum is assigned to each user. The simulation calculates the average spectrum allocation for each scenario by summing the allocated spectrum for all users and dividing it by the total number of users. Performance evaluation is done using metrics such as average throughput, outage probability, and spectrum allocation. The average throughput represents the average data rate achieved by the users, while the outage probability indicates the likelihood of a user experiencing a communication outage. The code likely involves simulations or analytical calculations to estimate these metrics for varying numbers of users. The results are then visualized through plots, allowing for a comparison of the performance of the different communication scenarios in [30].

### Simulation setup

To evaluate the performance of D2D communication in an outdoor urban environment, the simulation is initiated considering the parameters shown in Table 2

**Table 2:** Simulation Parameters.

Parameter	Value
Frequency	2.4 GHz
Simulation duration	30 seconds
Call duration	1 second
Sub-channel length	1 ms
Call requests per second	5 calls
Users (second scenario)	[0 - 150]
Max users base station	10
Users displacement	1 step / second
Users locations	Random
Channel bandwidth	180 kHz
Bandwidth	1 MHz
D2D association distance	10 m
Resource resharing distance	50 m
Pathloss exponent	2.5
Noise power	90 dBm
D2D Tx power	20 dBm
BS Tx power	20 dBm

These parameters are selected based on commonly used values for D2D and cellular communication, to enable realistic study the impact of user location, user density, and resource allocation on the performance of the D2D communication system in an outdoor urban environment. The rest of the parameters were quoted from [31].

**Fig 4:**
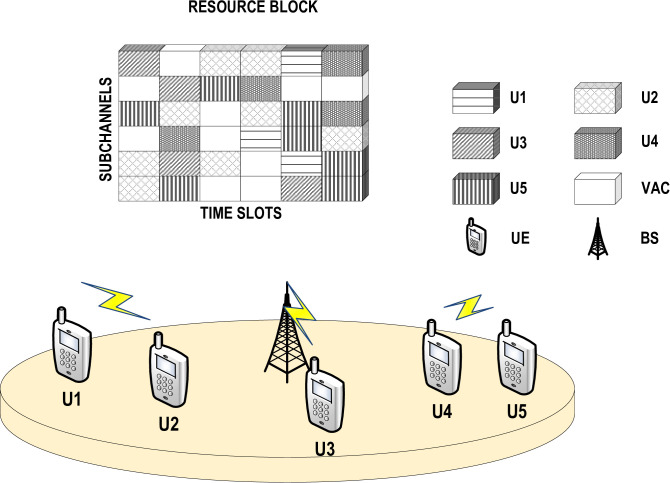
Simulation scenario.

Fig 4 shows the simplified experiment scenario whereby several users are spread around the coverage area. Each of these users is allocated some amount of subchannels based on the need and the availability of the resources. Subsequently, this will result some resources to be blocked for some users and some will be vacant as annotated with VAC in the Fig 4.

**Fig 5:**
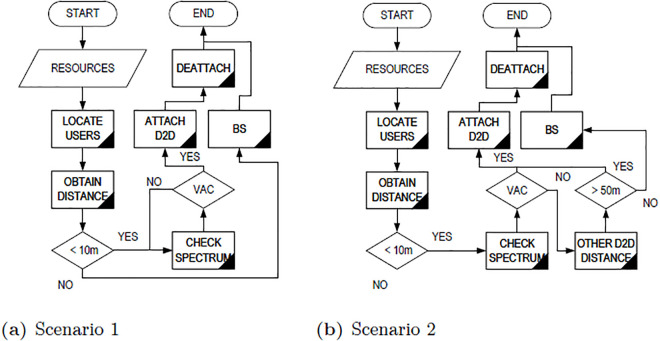
Simulation block diagram.

Fig 5 (a) shows the block diagram whereby the simulation runs through. After the distance between the users is calculated, any nearby users that are separated with a distance of less than 10 m, will establish D2D communication with any of the available resource.

The selection of 10 meters as the activation threshold for initiating D2D communication is motivated by the need to ensure strong signal quality and minimize path loss between paired devices. Within this short range, devices can reliably communicate using lower transmission power, which not only enhances energy efficiency but also reduces interference to surrounding users and the cellular base station. This aligns with findings in prior studies that identify sub-10m distances as optimal for proximity-based services and low-latency applications in dense urban environments [32].

The second scenario explained in Fig 5 (b) tries to minimize the outage probability that may result from fully occupying the resources and in case a sudden D2D attach request or base station request is received. This is solved by looking again into the distance matrix and select the resources used by the users that are located more than 50 m away to insure that the minimum interference separation distance is maintained.

On the other hand, the 50-meter threshold serves as a guard distance to allow resource reuse by other D2D users while minimizing co-channel interference. This distance is based on empirical observations in D2D studies and simulation environments, which show that beyond 50 meters, the likelihood of significant interference between D2D links drops substantially due to path loss and signal attenuation. By enforcing this spatial reuse rule, the system ensures better spectral efficiency without compromizing the quality of service for either the primary D2D link or nearby users.

**Fig 6:**
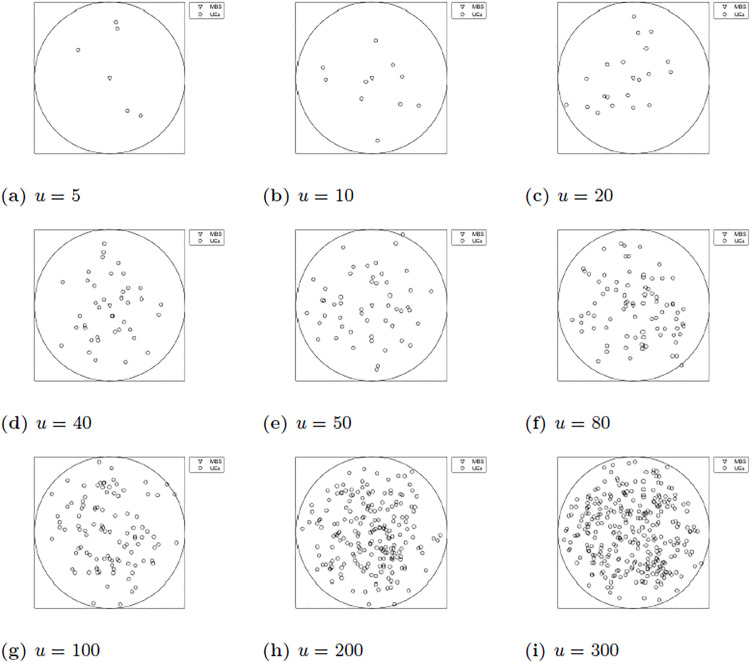
Simulation Area (Scenario 2).

Similarly, in the second scenario, users are spread randomly in the service area. However, the number of users keep changing after each iteration of steps. Fig 6 shows the service area for each user density. Moreover, and while the locations are completely random, each of the users moves one step in any direction for the total duration of the simulation.

The simulation considers various parameters such as the number of users, cell radius, D2D range, safe distance, bandwidth, noise power, path loss exponent, interference threshold, maximum transmit power, and maximum number of users the base station can handle simultaneously. The simulation begins by initializing arrays to store the results of each scenario, including the throughput, outage probability, and spectrum allocation percentage. It then proceeds to iterate over different numbers of users.

For the D2D scenario, the code generates random user locations within the cell and performs spectrum allocation and proximity checks to determine which users can communicate with each other. It calculates the throughput, outage probability, and spectrum allocation percentage for this scenario.

Initially, the users are randomly deployed in the service area which is considered to be as a microcell in these simulations. This process relies on positioning the microcell at Cartesian coordinates of (*x*_*Mc*_, *y*_*Mc*_) = (0,0), the procedure starts by generating a movement parameter that helps in identifying the direction for the users as shown in Equation 1


$$\begin{array}{c} t=0:0.0167:\rho \end{array}$$ (1)

where *t* is the total movements count for the simulation, and ρ is the simulation time. Following that, direction for the movement is generated randomly, i.e., 1 or −1 to represent the diredtion of movement

In the same manner, the movement parameter is obtained through the following equation:


$$\begin{array}{c} m=d\times \eta \end{array}$$ (2)

whereby η is a random number generated from the continuous uniform distribution between lower and upper endpoints. The upper endpoint is the step size that the user should move while the lower endpoint is the same value with a minus sign. It is also important to note that since the Cartesian coordinates are considered in this simulation, different movement parameters have to be obtained for the *x* and *y* coordinates to simulate the random movement in all directions.

The location can now be generated through the following equations:


$$\begin{array}{c} x_{cue}=x_{Mc}+l_{temp}\times cos\theta \end{array}$$ (3)

$$\begin{array}{c} y_{cue}=y_{Mc}+l_{temp}\times cos\theta \end{array}$$ (4)

whereby *l*_*temp*_ is a temporary location that can be generated using Equation 5


$$\begin{array}{c} l_{temp}=\sqrt{rand(1,cue)\times (R_{Mc}-t)} \end{array}$$ (5)

and θ is a random parameter that is generated by Equation 6.


$$\begin{array}{c} \theta =randn(1,cue)\times 2\pi \end{array}$$ (6)

Similarly, for the base station scenario, the code allocates spectrum to the closest users based on their distances to the base station. It calculates the throughput, outage probability, and spectrum allocation percentage.

In the hybrid scenario, the code combines the approaches of D2D and base station communication by allocating spectrum to both D2D users and base station users. It calculates the throughput, outage probability, and spectrum allocation percentage accordingly. It must also be noted that in the case of unavailability of resource, both users will communication through the normal base station resources.

#### User association.

Initially, when the simulation starts all users are associated with the Macrocell and that is done using an association array of the dimension of UE × UE. When the value of the array location is set to 0, means that the user is associated to the Macrocell while when it is set to 1, the two users are in D2D mode.

The association index α can be set based on the Equation 7:


$$\begin{array}{cc} \alpha (i,j) & =\;1\;\forall \;i\text{ and }j\;\in \text{UE}\\ s.t. & \\ & d_{i,j}\leq d_{establishment}\\ & vac\geq 1\;||\;d_{i,j}\geq d_{safe} \end{array}$$ (7)

### Performance metrics

This section provides some of the QoS metrics that will be considered to evaluate the performance of the D2D scenario.

#### Outage.

Signal outage may result in response to receiving high interference in comparison to the signal power of the desired signals, i.e., signal to interference ratio (SINR), inability to assign resources or not being able to receive any signals at all. In all cases, users experience signal starvation will not be able to establish communication, i.e., outage. The probability of such is the set of values between 0 and 1 whereby the system is said to be in complete outage and incapable of providing service to users if the probability equals to 1.

The outage as calculated by Equation (8) is bound by measuring the SINR *Prob*[*SINR* < *threshold*] of all users *U* and compare it to a threshold level that is set according to the QoS requirements as shown in Table 2.


$$\begin{array}{c} Outage=[\sum_{u\in U}(B+log_{2}[\frac{\frac{\sum_{u\in U}\sum_{k\in K}P_{u,k}^{t}G^{t}G_{u}^{r}\lambda }{(4\pi d_{t,u}{)}^{2}}}{\frac{\sum_{i\neq u\in U}\sum_{k\in K}P_{i,k}^{t}G^{t}G_{i}^{r}\lambda }{(4\pi d_{t,i}{)}^{2}}+\Gamma }]<threshold)U] \end{array}$$ (8)

whereby *u* is the user index, *i* is also the user index but different than *u*, the threshold value is set to 26.4 dBm [33], *G*^*t*^ is the transmitter’s gain, *G*^*r*^ is the receiver’s gain, *d*^*t*^ is the separation distance between the transmitter and the receiver, λ is the wavelength that is directly related to the frequency, and Γ is the noise figure.

#### Throughput.

Throughput refers to the measure of the amount of data that can be transmitted over a communication channel within a given time frame. It is a fundamental metric that quantifies the efficiency and capacity of a system to deliver data from a source to a destination. Throughput is typically expressed in bits per second (bps) or a related unit such as kilobits per second (Kbps) or megabits per second (Mbps).

#### Spectrum efficiency.

Spectrum efficiency is a measure of how efficiently the available frequency spectrum is utilized to transmit data or information. It quantifies the amount of information that can be reliably transmitted over a given bandwidth of the spectrum. Spectrum efficiency is typically expressed in terms of bits per second per hertz (bps/Hz) or a related unit such as kilobits per second per hertz (Kbps/Hz) or megabits per second per hertz (Mbps/Hz). It indicates the rate at which data can be transmitted over a specific frequency bandwidth. Higher spectrum efficiency means that more data can be transmitted within a given bandwidth, resulting in higher data rates or more simultaneous users. It is an important factor in optimizing the utilization of the limited frequency spectrum resources.

## Performance evaluation

**Fig 7:**
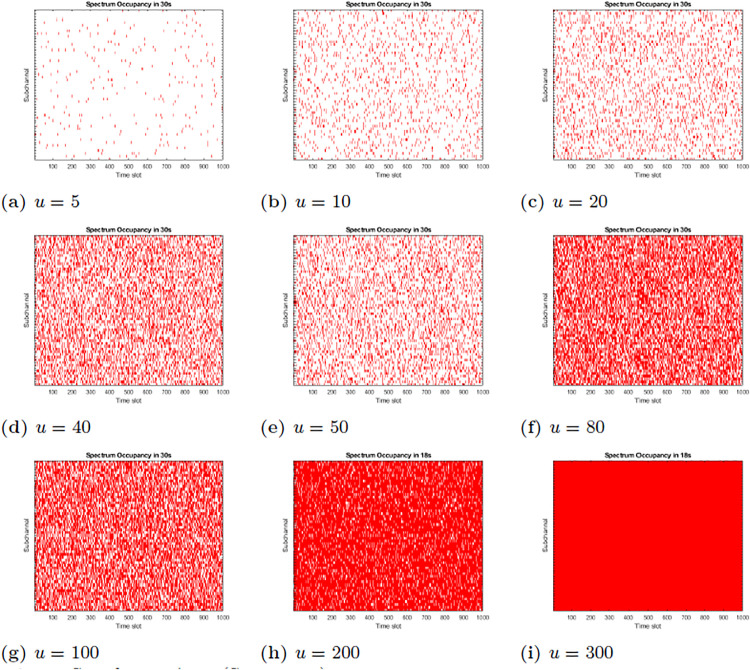
Simulation Area (Scenario 1).

This section presents the performance evaluations of the proposed system whereby the spectrum utilization results presented in a Fig 7, indicates that as the number of users increases in a D2D communication system, the utilization of the spectrum also increases.

This is evident in the figure as the y-axis represents the sub-channels, the x-axis represents the time slot, and the red dots represents the in-use sub-channel at the specific time-slot. It can also be understood that the spectrum utilization also increases and reaches its peak when the number of users is at its maximum.

However, as the number of users continues to increase, the system may eventually reach a point where there are no more vacant resources to be used, i.e., outage. Comparing by Fig 7 (a), (b) and (c) in which the number of users = 5, 10, and 200, respectively, it can be noticed that the probability of occupancy increases in relation to *u*. Nevertheless, the spectrum is not fully occupied. Similarly, Fig 7 (d), (e), and (f), the spectrum utilization expands with the increase of users.

Despite this, Fig 7 (d) for *u* = 40 shows that the spectrum is slightly over utilized in comparison to Fig 7 (e) for *u* = 50 which can be justified by the random distribution for the users’ call duration in the simulation’s spectrum function.

It is important to highlight that all users in this scenario are enabled to access the resources at the same time while allowing the user to initiate a call each second with a different call duration. Additionally, it could be understood from comparing Fig 7 (d) with 7 (f) that the available spectrum starts to decrease. Similarly in Fig 7 (g) and (h) whereby it can be seen that it is almost full and no additional resources are available for use typically due to the congestion in the network that can be caused by the high volume of user traffic. This also suggests that the system performance may be poor when the number of users is high which may lead to increased interference and reduced network capacity. Subsequently, The system outage reaches almost 100% when *u* = 300 and no further call requests can be processed.

**Fig 8:**
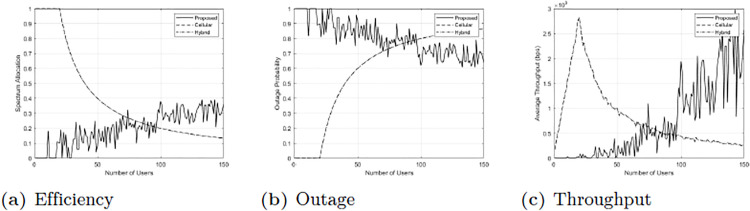
Simulation results.

On the other hand, the second scenario suggests that the same resources are to be accessed by distanced users to help utilize the use of the available resources and improve overall system performance in high-density scenarios. This can be noticed in Fig 8 which shows a considerable improvement in terms of the outage whereby it is suppressed to almost zero when 100 users are there. The outage probability only jumps to more than %100 if the users count jumps to about 400 users. This is also caused by the congestion of the network whereby if the number of the users and/or the call requests keep increasing considering the limited resources, the outage inflates noticeably.

The presented results in Fig 8 illustrate a comparative performance evaluation of three communication schemes namely Proposed, Cellular, and Hybrid, with respect to spectrum efficiency, outage probability, and average throughput in comparison with the number of users. These results provide critical insights into the scalability and robustness of the proposed D2D-enabled resource allocation approach in an urban wireless environment.

In Fig 8 (a), the proposed scheme demonstrates a gradual increase in spectrum utilization as user density grows, stabilizing around 30–40% allocation efficiency. This pattern suggests that the proposed mechanism exploits user proximity and spatial separation effectively, enabling dynamic reuse of radio resources and better scalability. In contrast, the cellular-only approach shows a rapid and a continuous decline in spectrum allocation efficiency, quickly falling below 20% as the number of users rises. This reflects the limitations of conventional cellular systems under high user load, where spectral congestion becomes a challenge. The hybrid approach performs slightly better than the cellular baseline but also trends downward after a certain threshold, indicating its inability to fully exploit spatial reuse in dense scenarios.

Fig 8 (b) depicts the outage probability in which the cellular scheme begins with low outage but deteriorates rapidly, reaching near-complete service failure (close to 100% outage) as the number of users exceeds 90. This can be explained by the insufficiency of cellular infrastructure in handling dense traffic without introducing significant communication failures. The hybrid scheme initially suffers from high outage at low user density which is potentially due to the overhead of switching between D2D and cellular modes. However, it stabilizes slightly below the cellular line in high-density scenarios. The proposed scheme, however, maintains a relatively consistent and lower outage probability throughout the user range. Despite minor fluctuations, it consistently outperforms the other approaches, highlighting its robustness and reliability in maintaining service availability under pressure.

Fig 8 (c) focuses on the average system throughput whereby the proposed scheme shows a clear advantage in scalability, with throughput steadily increasing as user density grows, peaking beyond 2 Gbps with over 140 users. This demonstrates that the scheme not only accommodates more users but also benefits from higher density by intelligently reusing resources. Conversely, the hybrid system achieves a sharp early peak around 30 users but then declines, indicating limited capacity to maintain high throughput in denser environments. The cellular system again shows a downward trajectory after a modest peak, reinforcing the inefficiency of traditional cellular models in supporting heavy traffic.

Together, these results underscore the superiority of the proposed approach in high-density, real-world scenarios. It balances spectral efficiency, minimizes outages, and supports scalable throughput, positioning it as a viable solution for future wireless systems where user demands and density are expected to surge dramatically.

It is important to state that the 50 meters that is selected as a safe separation distance was selected randomly to prove the concept. Besides this, the limitations of the simulation considered in this paper is the duration of the calls whereby all users have the same call duration without specifying the process of the calls variation. Additionally, the resource allocation process must by conditional to the availability of the user him/her self, i.e., if the user is in another call, the call should be rejected and the resources are to remain vacant.

When comparing the results of this work to those presented in [30], a significant divergence in the impact of D2D communication on network performance becomes evident whereby the expected outage probability for D2D communication remains below 5% for both uplink and downlink cases, showcasing a high level of reliability in D2D links. However, it also illustrates a relative degradation in CC scheme, where the presence of D2D interference; particularly in the downlink causes CC outage probability to expand for above 40%. This indicates a strong interference impact when spectrum is shared. In contrast, the results in Fig 8 reveal that the proposed D2D strategy not only reduces outage probability but also improves overall throughput and spectrum allocation efficiency compared to both cellular-only and hybrid approaches.

Although the interference cost on CC links can be managed, but the proposed approach appears to manage interference more effectively, maintaining relatively stable and lower outage probability across increasing user counts while maximizing throughput. This contrast suggests that distance-based or region-aware resource allocation, as employed above, may provide a more practical and scalable solution for coexistence of D2D and cellular links in dense user environments.

To replicate and validate the results experimentally, a controlled outdoor urban simulation can be configured to simulate the spatial distribution of users and the defined distance-based allocation thresholds (e.g., 10 m for D2D connectivity and 50 m for resource reuse). Performance metrics such as SINR, throughput, and outage probability can be measured under varying user densities and mobility patterns. Additionally, integration into a 5G environment provides valuable insights into real-world behavior, especially regarding network-assisted D2D communications and spectrum sharing under dynamic cellular loads.

## Conclusion

Given the growing prominence of D2D communication as a key enabler in the architecture of next-generation wireless networks, this work undertakes a comprehensive simulation-based performance evaluation under varied resource allocation strategies. The first scenario adopts a region-based segmentation, wherein users are spatially classified into inner and outer zones, with the spectral bandwidth accordingly partitioned to mitigate inter-region interference and enhance localized efficiency. The second scenario implements a proximity-aware resource allocation scheme, wherein radio resources are allocated to users within a 10-meter range and opportunistically reused by spatially distant users exceeding a 50 meter separation threshold, thereby promoting spectral reuse while managing interference constraints. The simulation environment is configured using parameters representative of typical urban macrocell deployments for both D2D and cellular communication. The study investigates the interplay between user topology, density variations, and allocation methodology, offering a comparative analysis of the two scenarios to elucidate their respective impacts on system throughput, interference levels, and overall communication reliability within the D2D paradigm.

It is worth mentioning that the simulation assumes that the users are distributed uniformly in the covered area, and this might not be the case in a real-world scenario, which could lead to a limitation in the accuracy of the results. Additionally, the simulation only considers a 2-dimensional area and does not take into account the height of the users or obstacles, which can affect the signal propagation and system performance. Furthermore, The simulation also assumes that the channel is fixed and does not take into account the fading and shadowing effect, which can have a significant impact on the system’s performance. Also, to streamline the simulation process, we adopted simplifying assumptions such as fixed call durations and linear user mobility. While these choices help in isolating core performance metrics, they limit the generalizability of the results. In real-world scenarios, call durations fluctuate based on application context (e.g., voice vs. video), directly impacting spectrum usage and interference patterns. Additionally, user movement is rarely linear; it is often influenced by environment dynamics, user behavior, and mobility models such as random waypoint or Gauss-Markov. These assumptions may cause the simulation to overestimate reliability and efficiency under ideal conditions, thus emphasizing the need for future work that incorporates more realistic usage models.

## Supporting information

S1 File Support info. (PDF)
