# Supplementary material for: Distance-based Device-To-Device outage reduction for 5G wireless systems
Source: PLoS One. 2026 Jun 15;21(6):e0335050. doi: 10.1371/journal.pone.0335050 (PMC13268130; doi:10.1371/journal.pone.0335050)
Supplement: S1 File — (PDF) [file pone.0335050.s001.pdf]

```
close all;


N_users = 150;
cell_radius = 300;
d2d_range = 10;
safe_distance = 50;
bandwidth = 10e6;
noise_power = -174 + 10*log10(bandwidth);
path_loss_exponent = 3;
interference_threshold = -100;
max_power = 23;
max_bs_users = 20;

throughput_results_d2d = zeros(N_users, 1);
outage_results_d2d = zeros(N_users, 1);
spectrum_allocation_results_d2d = zeros(N_users, 1);

throughput_results_bs = zeros(N_users, 1);
outage_results_bs = zeros(N_users, 1);
spectrum_allocation_results_bs = zeros(N_users, 1);

throughput_results_hybrid = zeros(N_users, 1);
outage_results_hybrid = zeros(N_users, 1);
spectrum_allocation_results_hybrid = zeros(N_users, 1);


for num_users = 1:N_users
    user_locations = cell_radius * rand(num_users, 2);
```

```
spectrum_d2d = zeros(num_users, 1);
spectrum_available_d2d = ones(num_users, 1);

for i = 1:num_users
    for j = i+1:num_users
        distance = norm(user_locations(i,:) - user_locations(j,:));

        if distance <= d2d_range
            if spectrum_available_d2d(i) && spectrum_available_d2d(j)
                spectrum_d2d(i) = 1;
                spectrum_d2d(j) = 1;
                spectrum_available_d2d(i) = 0;
                spectrum_available_d2d(j) = 0;
            end
        end
    end
end

throughput_d2d = zeros(num_users, 1);
for i = 1:num_users
    if spectrum_d2d(i) == 1
        distance_bs = norm(user_locations(i,:));

        path_loss = 10*path_loss_exponent*log10(distance_bs);
        received_power = max_power - path_loss;
        interference = 0;
        for k = 1:num_users
            if k ~= i && spectrum_d2d(k) == 1
```

```
            interference_distance = norm(user_locations(i,:) - user_locations(k,:));
            interference_path_loss = 10*path_loss_exponent*log10(interference_distance);
            interference = interference + max_power - interference_path_loss;
          end
        end

        sinr = received_power - noise_power - interference;
        throughput_d2d(i) = bandwidth * log2(1 + 10^(sinr/10));
    end
end
outage_d2d = sum(spectrum_d2d == 0) / num_users;
spectrum_allocation_d2d = sum(spectrum_d2d) / num_users;
throughput_results_d2d(num_users) = mean(throughput_d2d);
outage_results_d2d(num_users) = outage_d2d;
spectrum_allocation_results_d2d(num_users) = spectrum_allocation_d2d;

spectrum_bs = zeros(num_users, 1);
spectrum_available_bs = ones(num_users, 1);

num_bs_users = min(num_users, max_bs_users);

[~, sorted_indices] = sort(vecnorm(user_locations, 2, 2));
spectrum_bs(sorted_indices(1:num_bs_users)) = 1;
spectrum_available_bs(sorted_indices(1:num_bs_users)) = 0;

throughput_bs = zeros(num_users, 1);
for i = 1:num_users
    if spectrum_bs(i) == 1
        distance_bs = norm(user_locations(i,:));
```

```
        path_loss = 10*path_loss_exponent*log10(distance_bs);
        received_power = max_power - path_loss;
        interference = 0;
        for k = 1:num_users
            if k ~= i && spectrum_bs(k) == 1
                interference_distance = norm(user_locations(i,:) - user_locations(k,:));
                interference_path_loss = 10*path_loss_exponent*log10(interference_distance);
                interference = interference + max_power - interference_path_loss;
            end
        end

        sinr = received_power - noise_power - interference;
        throughput_bs(i) = bandwidth * log2(1 + 10^(sinr/10));
    end
end

outage_bs = sum(spectrum_bs == 0) / num_users;
spectrum_allocation_bs = sum(spectrum_bs) / num_users;
throughput_results_bs(num_users) = mean(throughput_bs);
outage_results_bs(num_users) = outage_bs;
spectrum_allocation_results_bs(num_users) = spectrum_allocation_bs;

spectrum_hybrid = zeros(num_users, 1);
spectrum_available_hybrid = ones(num_users, 1);

num_hybrid_users = min(num_users, max_bs_users);
spectrum_hybrid(sorted_indices(1:num_hybrid_users)) = 1;
spectrum_available_hybrid(sorted_indices(1:num_hybrid_users)) = 0;
for i = 1:num_users
```

```
    if spectrum_hybrid(i) == 0
        for j = 1:num_users
            if j ~= i && spectrum_hybrid(j) == 1
                distance = norm(user_locations(i,:) - user_locations(j,:));
                if distance <= d2d_range
                    if spectrum_available_hybrid(i) && spectrum_available_hybrid(j)
                        spectrum_hybrid(i) = 1;
                        spectrum_hybrid(j) = 1;
                        spectrum_available_hybrid(i) = 0;
                        spectrum_available_hybrid(j) = 0;
                        break;
                    end
                end
            end
        end
    end
end

throughput_hybrid = zeros(num_users, 1);
for i = 1:num_users
    if spectrum_hybrid(i) == 1
        distance_bs = norm(user_locations(i,:));
        path_loss = 10*path_loss_exponent*log10(distance_bs);
        received_power = max_power - path_loss;
        interference = 0;
        for k = 1:num_users
            if k ~= i && spectrum_hybrid(k) == 1
                interference_distance = norm(user_locations(i,:) - user_locations(k,:));
                interference_path_loss = 10*path_loss_exponent*log10(interference_distance);
```

```
                interference = interference + max_power - interference_path_loss;
            end
        end

        sinr = received_power - noise_power - interference;
        throughput_hybrid(i) = bandwidth * log2(1 + 10^(sinr/10));
    end
end

outage_hybrid = sum(spectrum_hybrid == 0) / num_users;

spectrum_allocation_hybrid = sum(spectrum_hybrid) / num_users;
throughput_results_hybrid(num_users) = mean(throughput_hybrid);
outage_results_hybrid(num_users) = outage_hybrid;
spectrum_allocation_results_hybrid(num_users) = spectrum_allocation_hybrid;
end

figure;
x = 1:N_users;
plot(x, throughput_results_d2d, 'k-', 'LineWidth', 1);
hold on;
plot(x, throughput_results_bs, 'k--', 'LineWidth', 1);
plot(x, throughput_results_hybrid, 'k-.', 'LineWidth', 1);
xlabel('Number of Users');
ylabel('Average Throughput (bps)');
legend('Proposed', 'Cellular', 'Hybrid');
grid on;

figure;
```

```
plot(x, outage_results_d2d, 'k-', 'LineWidth', 1);
hold on;
plot(x, outage_results_bs, 'k--', 'LineWidth', 1);
plot(x, outage_results_hybrid, 'k-.', 'LineWidth', 1);
xlabel('Number of Users');
ylabel('Outage Probability');
legend('Proposed', 'Cellular', 'Hybrid');
grid on;

figure;
plot(x, spectrum_allocation_results_d2d, 'k-', 'LineWidth', 1);
hold on;
plot(x, spectrum_allocation_results_bs, 'k--', 'LineWidth', 1);
plot(x, spectrum_allocation_results_hybrid, 'k-.', 'LineWidth', 1);
xlabel('Number of Users');
ylabel('Spectrum Allocation');
legend('Proposed', 'Cellular', 'Hybrid');
grid on;
```
